# How do Children with Intellectual Disabilities Empathize in Comparison to Typically Developing Children?

**DOI:** 10.1007/s10803-024-06340-3

**Published:** 2024-04-12

**Authors:** Poline Simon, Nathalie Nader-Grosbois

**Affiliations:** Chair Baron Frère in special education, Psychological Sciences Research Institute, UCLouvain, Louvain-la-Neuve, Belgium

**Keywords:** Affective empathy, Cognitive empathy, Behavioral empathy, Developmental stages, Intellectual disabilities, Down syndrome

## Abstract

**Objectives:**

Two studies were conducted to better understand how children with intellectual disabilities (ID) empathize with the feelings of others during social interactions. The first study tested hypotheses of developmental delay or difference regarding empathy in 79 children with ID by comparing them with typically developing (TD) children, matched for developmental age or chronological age. The second study examined specific aspects of empathy in 23 children with Down syndrome (DS), compared with 23 nonspecific ID children, matched for developmental age, and TD children, matched for developmental age or chronological age.

**Method:**

An empathy task was administered to the children while their parents completed the French versions of the Empathy Questionnaire and the Griffith Empathy Measure.

**Results:**

The first study showed that ID children showed delayed empathy development but were perceived by their parents as deficient in cognitive empathy. The second study showed that DS children were perceived as being more attentive to the feelings of others than TD children and non-specific ID children, matched for developmental age, and as having affective empathy that was similar to that of TD children matched for chronological age.

**Conclusion:**

These studies have drawn attention to delays or differences in different dimensions of empathy in children with ID and DS, which need to be taken into account in interventions.

**Supplementary Information:**

The online version contains supplementary material available at 10.1007/s10803-024-06340-3.

## Developmental and Multidimensional Conceptions of Empathy in TD Children

Recent studies have proposed two conceptualizations that can be used to examine the specific characteristics of empathic skills in typically and atypically developing children. The first of these is a developmental approach. According to Hoffman’s (2000, p.7) development model, empathy involves one’s emotional state mirroring the emotional state of another person and is primarily influenced by the situation experienced by the other person rather than the situation of the empathizing individual. Hoffman’s (1987) earlier developmental model consisted of four stages from infancy to adulthood. The first stage, known as emotion contagion, occurs during the first year of life and involves automatic reactions to others’ emotional responses, including gestures, behaviors, and vocalizations (Hatfield et al., 1993). In the second stage, labeled as attention to others’ feelings, one-year-olds expressed reduced distress regarding others’ emotions. But, they become more attentive to the emotional expressions of people around them. At the third stage, as children enter their second year, they develop concern for others and engage in prosocial behaviors such as sharing, helping, and comforting. From six years old, the final stage, known as empathy for others’ conditions, involves experiencing empathy for the broader life circumstances of individuals, such as those facing challenges like cancer. In 2000, Hoffman updated this model and developed a five-stage model as follows: (1) reactive newborn crying (infants cry when they hear another infant cry), (2) egocentric empathic distress (children try to relieve their own distress), (3) quasi-egocentric empathic distress (children try to relieve the distress of others through behaviors that are helpful to themselves), (4) verified empathic distress (children adapt their prosocial behaviors to the needs of others), and (5) empathic distress beyond the situation (equivalent to empathy for the life condition of others).

In multi-dimensional conceptions, empathy is defined as an affective response which stems from the understanding of others’ emotional situation (Eisenberg et al., 2006) and has two major components. First, affective empathy refers to the capacity to share others’ emotional experiences and feel others’ congruent emotions in terms of intensity and valence (Decety & Holvoet, 2021). Second, cognitive empathy is defined as the understanding of others’ emotions (Decety, 2015). This capacity allows people to intentionally take others’ perspective and understand the reasons for others’ emotions (Decety & Holvoet, 2021). According to Heyes (2018), affective empathy leads to automatic responses to others’ emotions, whereas cognitive empathy is based on controlled responses. Some authors add behavioral empathy to designate behaviors displayed toward others, resulting from affective and cognitive empathy (Tamayo et al., 2016). These empathic behaviors could be socially appropriate for the target (e.g., comforting or helping) or not (e.g., thwarting or disturbing) (Heyes, 2018). In a radical behaviorist view, empathic behaviors could be perceived as reinforcement for individuals, which stem from the understanding of the emotional state of other person (Melton et al., 2023). In their paper, Neumann et al. (2015) have index measures of empathy which evaluate affective, cognitive and behavioral empathy.

Beyond the literature exploring empathy in TD children at preschool and school age, several authors have explored the links between empathy and other skills (e.g., Theory of Mind, emotion regulation, social skills, etc.). For example, affective and cognitive empathy, as well as prosocial actions, are positively linked to Theory of Mind (ToM) (Bensalah et al., 2016; Brown et al., 2017) and emotion regulation (Laghi et al., 2018; Lee et al., 2016; Lucas-Molina et al., 2018). Moreover, empathic children have better social skills (Findlay et al., 2006; Simon & Nader-Grosbois, 2021), are more accepted by their peers (Braza et al., 2009) and show fewer behavior problems (Simon & Nader-Grosbois, 2021). Even though the literature about empathy in TD children continues to expand, studies on children with ID are still not widespread (e.g. Simon & Nader-Grosbois, 2023 for a review). Therefore, the purpose of this research (including two studies) was to explore the empathic skills in children with ID, compared to TD children, matched for chronological or developmental age. The first study attempts to highlight which differences could be identified in empathy in children with and without intellectual disabilities. The second study aims to examine the empathic skills of children with genetic syndrome (notably Down syndrome) in comparison to TD children with a preschool developmental age (3 to 6 years).

## Hypotheses Regarding Development of Children with ID

The development of children with ID is commonly characterized in reference to developmental *versus* difference postulates, synthesized by Zigler (1969). On the one hand, the *developmental hypothesis* states that children with ID follow the same developmental stages and present the same cognitive structures as TD children, matched for developmental age, but the speed of acquiring skills and making the transitions from one stage to another is slower. Children with ID may not reach the more complex developmental stages. On the other hand, the *difference hypothesis* refers to a deficit or positive difference in the skills of children with ID regarding their acquisition sequences, stage transitions, cognitive structures, or reasoning, compared to TD children matched for developmental age. These postulates have been extended to other domains, including emotional and social skills (Zigler, 1969). Moreover, an ecological approach has been recommended to examine children with ID’s skills in daily situations (Hodapp et al., 1998). Beyond these two hypotheses, Inhelder (1963) postulated that children with ID exhibit a *viscosity of reasoning*, expressed by oscillations between different levels of complexity in difficult transitions. Paour (1992) suggested a *cognitive subfunctioning*, stating that children with ID have difficulties in mobilizing their cognitive skills because of emotional, contextual, and motivational factors. These last hypotheses could also account for specific features of emotional and social (in)abilities in these children.

## Empathy in Children with ID and Their Emotional and Social Skills

Children with ID present a developmental delay in their understanding of causes and consequences of the four basic emotions (Baurain & Nader-Grosbois, 2013; Thirion-Marissiaux & Nader-Grosbois, 2008b), and a deficit in understanding beliefs and intentions (Jacobs et al., 2020; Thirion-Marissiaux & Nader-Grosbois, 2008a) as well as in social information processing (Jacobs et al., 2020). These specific aspects of social cognition are related to difficulties in their emotion regulation (Baurain & Nader-Grosbois, 2013). In addition, studies have highlighted a deficit in prosocial behaviors and difficulties in social adjustment with peers (Guralnick et al., 2006; Zion & Jenvey, 2006). Children with ID are also at greater risk of internalized and/or externalized behavior problems (Baker et al., 2002, 2010; Merrell & Holland, 1997; Nader-Grosbois et al., 2013). Because of these difficulties, it seemed relevant to examine how children with ID (without specific etiology or with Down syndrome) empathize at preschool and school age and to identify their strengths or weaknesses in empathy stages (emotion contagion, attention to others’ feelings and prosocial actions) or components (affective, cognitive and behavioral empathy). Given their difficulties in social adjustment and their risk of victimization, it could be useful to gain a better understanding of their empathy with others’ emotions, with the aim of promoting appropriate empathic reactions and subsequently social acceptance and inclusion. Although some studies have explored children with ID’s empathic skills (e.g., Corona et al., 1998; Sigman et al., 1992), none of them has effectively tested delay *versus* difference hypotheses or been based on recent conceptions of empathy. However, previous comparative studies have provided some information about empathic skills of children with ID but the results did not converge through studies (see Table 1 for a summary). For example, Sigman et al. (1992) did not find any difference between children with ID and TD children matched for developmental age (mean age of 2 years) in their empathic reactions. Based on an observational design, both groups paid more attention to and were more concerned about an adult expressing distress, fear, or discomfort than was the case with children with autism spectrum disorder (ASD). Conversely, Corona et al. (1998) demonstrated that, even when children were paired for developmental age (mean age of 2 years), the group with ID showed a weaker level of empathy as recorded by a distress-inducing observation measure. Merrell et al. (1992) showed that children with ID were perceived by teachers, as measured through a questionnaire about social skills including empathy, as less empathic than TD children, paired for chronological age (between 5 and 13 years old). The same results were found in the study of Dyck et al. (2001) when empathic skills of 12-year-old children with ID were compared to those of TD children matched for chronological age, based on performance-based measures. However, these studies considered empathy as a global unitary construct. There was no indication of children’s skills in terms of developmental stages or components of empathy. Accordingly, the measures used in these studies did not consider recent multidimensional models of empathy, and the indicators used differed from study to study, making it difficult to draw any conclusion. Few studies have focused directly on individuals with ID, and this group has mainly been considered as a comparison sample in studies focusing on individuals with ASD. Moreover, matching criteria have differed between studies. Some studies have compared children with ID and other groups of children in terms of chronological age, implying indirectly the benefit from their multiple life experiences. Conversely, some researchers have chosen to match children with ID and the comparison groups for developmental age, ensuring a similar cognitive level in the children. To test whether there is a delay or a difference (whether a deficit or a positive difference) in the empathic skills of children with ID, studies need to assess the different stages or dimensions of empathy in order to compare their abilities with those of TD children, matched for developmental and chronological age.

**Table 1:** Summary of results of studies on empathy in children with ID and DS

Studies about empathy in children with intellectual disabilities					
Authors	Samples	Matching	Age range	Measures	Results
Sigman et al. (1992)	Children with ID TD children Children with ASD	Developmental	Mean of 2 years	Observational design	Children with ID paid attention expressed distress as much as TD children
Corona et al. (1998)	Children with ID TD children	Developmental	Mean of 2 years	Observational design	Children with ID have a weaker level of empathy than TD children
Merrell et al. (1992)	Children with ID TD children	Chronological	Between 5 and 13 years old	Hetero-reported questionnaire	Children with ID are perceived as having a weaker level of empathy than TD children
Dyck et al. (2001)	Children with ID TD children	Chronological	Mean of 12 years old	Performance-based measure	Children with ID have lower performance in empathy than TD children

Studies about empathy in children with Down syndrome					
Authors	Samples	Matching	Age range	Measures	Results
Plesa-Skwerer and Tager-Flusberg (2016)	Children with DS Children with Williams syndrome TD children	Chronological	Between 2 ;8 and 5;8 years old	Observational design	Children with DS have more nonverbal response to adult’s distress
Kasari et al. (2003)	Children with DS Children with ID TD children	Developmental	Mean of 4 years	Observational design Performance-based measure	In observational situation, children with DS look longer and offer more reassurance than TD children.
Hudry and Slaughter (2009)	Children with DS TD children Children with ASD	Chronological	Between 2 and 6 years	Hetero-reported questionnaire	Children with DS are less preoccupied than TD children

## Empathy in Children with Down Syndrome

It is interesting to know if a specific genetic syndrome, notably Down syndrome (DS), plays a role in empathy development, to identify potential specific characteristics in social cognition and to give guidelines to support their development from as early a stage as possible. Few studies (see Table 1 for a summary) have investigated empathy in children with DS. While some researchers have reported that children with DS present more non-verbal responses to adults’ distress (Plesa-Skwerer & Tager-Flusberg, 2016) or that they are less preoccupied (Hudry & Slaughter, 2009) than TD children paired for chronological age, no study has examined empathy in children with DS in terms of delay versus difference hypotheses. Only Kasari et al. (2003) matched children with DS and TD children according to their developmental age: they found that children with DS looked at the experimenter for longer and offered more reassurance than TD children. These studies about children with DS have also approached empathy as a unidimensional construct, without differentiating between distinct stages or components. Consequently, a deeper understanding of the empathic skills of children with DS could provide a better insight into whether they express over-empathy towards strangers, experience intense emotional contagion, understand others’ emotions, or are able to act prosocially, in social situations likely to elicit empathy. The matching group varied also in these studies: some authors paired children with DS with TD children according their developmental age (e.g., Kasari et al., 2003), and others according to their chronological age (e.g., Plesa-Skwerer & Tager‐Flusberg, 2016). No study carried out double matching to check developmental or difference hypotheses of empathy in children with DS or to compare them with children presenting other etiologies. Concerning the measures, observational design was commonly applied to assess empathy, but indicators varied from one study to another. For example, Kasari et al. (2003) were interested in emotions, prosocial behaviors, and gaze at adults, while Plesa-Skwerer and Tager‐Flusberg (2016) observed verbal and non-verbal reactions, facial expressions and global empathic reactions. Hudry and Slaughter (2009) used a hetero-reported questionnaire, presenting five emotional situations where parents had to indicate children’s reactions in terms of empathic concern, facial expressions, comforting and active play. An adapted version of the Affective Situations Test for Empathy (Feshbach & Roe, 1968), giving a global score, was administered to children in the study conducted by Kasari et al. (2003). All these differences in methodology impact the generalizability of the results.

Given the lack of studies using a multi-method and a double matching for chronological and developmental ages to identify empathy profiles in children with ID, including children with DS, two studies were conducted to test developmental delay *versus* difference hypotheses. The first one aimed to apprehend empathic skills in children with nonspecific ID, while the second study explored whether the development of empathic skills varied depending on the etiology of ID, notably DS and nonspecific ID.

## Study 1

### Method

#### *Objectives*

The first study compared empathic skills in children with mild to moderate ID and TD children matched for developmental or chronological age. Two approaches were used: (1) a developmental approach referring to the progression in stages of Hoffman’s (1987) model to explore if children with ID presented difficulties in each empathy development stage and (2) a multi-dimensional approach including affective, cognitive and behavioral empathy. Regarding the findings of the literature (e.g. Corona et al., 1998, Dyck et al., 2001; Sigman et al., 1992), it was hypothesized that children with ID have more emotion contagion but pay less attention to others’ feelings and prosocial actions as well as having fewer affective, cognitive, and behavioral empathic skills than TD children matched for chronological age. However, a developmental delay was expected regarding their affective and cognitive empathy (in line with Nader-Grosbois et al., 2013; Sigman et al., 1992), and it was expected that a deficit in behavioral empathy (based on Zion & Jenvey, 2006) would be observed when they were matched for their developmental age with TD children.

#### *Participants*

Three groups of children were recruited for this study. The first one consisted of 79 children with ID (35 girls and 44 boys), aged from 5 to 14 years, with a preschool developmental age range from 2,75 to 7 years), with reference to the inclusion criteria (see Table 2 for mean) (group with ID). They had been diagnosed by a psychologist (not included in this research) as having mild to moderate IDs (IQ between 50 and 70), according to AAIDD (American Association on Intellectual and Developmental Disabilities, 2011) and DSM-5-TR (Diagnostic and Statistical Manual of Mental Disorders, 2022) criteria. Children with an associated diagnosis of autism spectrum disorder were excluded. Concerning the socioeconomic status of the family, Table 3 shows means and percentages for parents’ educational level and family income for all samples. Eight mothers and 9 fathers of these children with ID had received special education. The information about educational level was missing for 26 mothers and 33 fathers. In terms of family income, the mean value of the sample corresponded to a monthly income of 2000–2500 euros.

**Table 2:** Descriptive statistics of Study 1

	ID	TD-DA	TD-CA		
	M (ET)	M (ET)	M (ET)	F	η_p_^2^
N (% of boys)	79 (55.7%)	92 (51.1%)	75 (52.6%)		
Chronological age (in years)	9.86 (2.26)	4.01 (0.923)	9.57 (2.00)		
Developmental age (in years)	4.90 (1.15)	4.61 (1.067)	/		
Language quotient (total)	0.69 (0.16)	0.70 (0.14)	/	2.625	0.016
Language Expression quotient	0.58 (0.25)	0.67 (0.19)	/	6.721*	0.040
Language Understanding quotient	0.77 (0.21)	0.77 (0.14)	/	0.006	0.000
***Hetero-reported measure***					
EmQue-vf - Emotion contagion (max = 4)	2.02 (0.45)	1.94 (0.39)	/	2.287	0.013
EmQue-vf - Attention to others’ feelings (max = 4)	2.72 (0.44)	2.80 (0.34)	/	1.220	0.007
EmQue-vf - Prosocial actions (max = 4)	2.45 (0.59)	2.23 (0.42)	/	13.190***	0.072
GEM-vf - Affective empathy (max = 4)	0.96 (0.95)	0.97 (0.87)	1.59 (1.85)	12.669***	0.094
GEM-vf - Cognitive empathy (max = 4)	0.39 (1.07)	0.81 (1.03)	1.68 (1.61)	34.254***	0.160
***Performance-based measure***					
Empathy task - Affective empathy (max = 8)	5.81 (1.86)	4.57 (2.26)	/	14.668***	0.081
Empathy task - Cognitive empathy (max = 8)	4.37 (2.27)	3.82 (2.48)	/	2.222	0.013
Empathy task - Behavioral empathy (max = 8)	3.85 (2.35)	3.51 (2.61)	/	0.791	0.005

*Notes* TD-DA = TD children matched for developmental age; TD-CA = TD children matched for chronological age; ID = children with ID; ***p < .001.

**Table 3:** Demographics characteristics of participants’ families

	ID		TD-DA		TD-CA	
	M (SD)	%	M (SD)	%	M (SD)	%
*Mothers’ educational level*	6.23 (2.35)		7.50 (1.63)		6.95 (1.92)	
≤ Vocational qualification		26.4%		4.9%		12.3%
High school qualification		28.3%		24.4%		24.7%
Bachelor’s degree		18.9%		29.2%		30.1%
Master’s degree		22.6%		34.2%		31.5%
PhD		3.8%		7.3%		1.4%
*Fathers’ educational level*	5.67 (2.70)		6.77 (1.80)		6.87 (1.88)	
≤ Vocational qualification		41.3%		14.6%		15.5%
High school qualification		23.9%		40.3%		28.2%
Bachelor’s degree		8.7%		15.8%		22.5%
Master’s degree		17.4%		25.6%		33.8%
PhD		8.7%		3.7%		/
*Family income*	5.51 (2.47)		7.88 (2.70)		8.89 (3.52)	

The second group consisted of 92 TD preschoolers, matched for developmental age (TD-DA group). A developmental age equivalent to 3 to 6 years was used as an inclusion criterion, as well as the absence of developmental delay. No difference concerning developmental age was reported between the group with ID and the TD-DA group (mean difference = 0.290, *p* = .10). For these two samples of children, one-way ANOVA showed that children with ID and TD children presented no difference in their levels of global language (*F* = 2.625, *p =* .107, *η*_*p*_^2^ = 0.016). However, through a one-way MANOVA (Pillai’s *F* = 3.995, *p* = .021, *η*_*p*_^2^ = 0.047), a slight difference appeared in language expression (*F* = 6.721, *p =* .01, *η*_*p*_^2^ = 0.04), stipulating that children with ID had lower skills to express themselves verbally. No difference in language comprehension was reported (*F* = 0.006, *p* = .938, *η*_*p*_^2^ = 0.000).

Concerning the family’s socioeconomic status, the educational level information was missing for 10 mothers and 10 fathers. In terms of family income, the mean value of the sample corresponded to a range of 3000–3500 euros per month.

The third group consisted of 75 TD children, matched for chronological age (TD-CA group). A chronological age of 4 to 14 years was an inclusion criterion as well as the absence of developmental delay. No difference concerning chronological age was reported between the group with ID and the TD-CA group (mean difference = 0.312, *p* = .846). Concerning the family’s socioeconomic status, educational level information was missing for 2 mothers and 4 fathers. In terms of family income, the mean value of the sample corresponded to a range of 3500–4000 euros per month.

With regard to the socio-economic status of families, chi-square analysis showed that the three groups of children differed significantly according to the level of education of mothers (χ^2^ = 43.830, df = 18, *p* < .001) and fathers (χ^2^ = 56.902, df = 18, *p* < .001) and also according to family incomes (χ^2^ = 81.835, df = 28, *p* < .001). Descriptive analysis showed that families of children with ID had a lower socio-economic status than both groups of TD children.

#### *Measures*

##### Son-R (Tellegen & Laros, 2009)

This non-verbal scale was used to estimate the developmental age of children to match TD children and children with ID. This measure allowed to assess cognitive skills of children aged between 2,5 and 7 years (chronological or developmental). Through six subscales, children’s performance (puzzle, mosaic and drawing) and reasoning (category, analogy and situations) skills were assessed, and a developmental age could be calculated thanks to raw scores. This evaluation ensured that children displayed a preschool developmental age and therefore met the criteria for inclusion.

##### Evaluation of Oral Language (ELO, Khomsi, 2001)

This measure consisted in evaluating the language level of children with ID and TD children, matched for developmental age. Through six subscales, a quotient score (from 0 to 1) related to comprehension and expression can be calculated.

##### Empathy Questionnaire – French Version (EmQue, Rieffe et al., 2010; EmQue-vf, Simon et al., 2023)

This questionnaire has been validated in French for assessing parents’ perception of empathy in children aged from 3 to 6 years. On a four-point Likert scale from “never” (1) to “always” (4), mothers and fathers indicated how frequently children’s empathic reactions and/or behaviors had occurred in the last two months. Conforming to the original version, the EmQue-vf was composed in three levels, referring to the first stages of the developmental model of (Hoffman, 1987, 2000): (1) emotion contagion (4 items, “*When another child cries, my child gets upset too”*), (2) attention to others’ feelings (6 items, “*When another child is angry, my child stops his own play to watch”*) and (3) prosocial actions (4 items, *“When another child starts to cry, my child tries to comfort him/her”).* In the French validation, reliability is adequate for Attention to other’s feelings (Cronbach’s α = 0.78) and Prosocial actions (Cronbach’s α = 0.81) but limited for Emotion contagion (Cronbach’s α = 0.65). In the present study, Cronbach’s alpha varied from 0.75 to 0.82 for TD children and from 0.73 to 0.85 for children with ID.

##### Griffith Empathy Measure – French Version (GEM, Dadds et al., 2008; GEM-vf, Nader-Grosbois & Simon, 2023)

This questionnaire, adapted from the Bryant Index of Empathy for Children and Adolescents (Bryant, 1982), evaluates parents’ perception of affective and cognitive empathy in their children aged from 4 to 16 years in the original version (Dadds et al., 2008) and from 3 to 12 years in the French version (Nader-Grosbois & Simon, 2023). Among the different hetero-reported measures of empathy, the GEM-vf was chosen for its wide range of age which corresponded to the chronological and developmental age of children in the different groups. Through 17 items, mothers and fathers separately had to rate their degree of agreement concerning their children’s behaviors and reactions to others’ emotions on a nine-point Likert scale, ranging from “strongly disagree” (-4) to “strongly agree” (4). Of the 17 items, 13 concerned affective empathy (e.g., *“It makes my child sad to see another child who can’t find anyone to play with”*) and 4 concerned cognitive empathy (*“My child rarely understands why other people cry”*). In the validation of the French version, Cronbach’s alpha was 0.78 for cognitive empathy and 0.62 for cognitive empathy. In the present study, internal consistency varied from 0.57 to 0.78 for TD children matched for developmental age, from 0.68 to 0.83 for TD children matched for chronological age and from 0.67 to 0.83 for children with ID.

##### Empathy Task (Bensalah et al., 2016)

Through eight stories (four with a boy and four with a girl), affective, cognitive and behavioral empathy were assessed. Each story featured children in an emotional situation (two stories for each basic emotion - happiness, sadness, fear, and anger), and the participants were asked several questions (1 point each). First, to ensure that the children understood the story, they were asked to retell it. If children were not able to describe and explain the story, the following questions were not asked. In the samples of this present study, all children understood the eight stories. Second, to assess affective empathy, they were asked to identify the emotion they themselves felt when hearing the story. At this point, the examiner could check whether the participants felt the same emotion as the character or not. Third, cognitive empathy was evaluated by asking the participants why they felt this kind of emotion. Finally, to apprehend behavioral empathy, the participants were asked what they would do if they were with the character. For each subscale, participants could obtain a maximum of 8 points. However, points for cognitive and behavioral empathy were only counted if participants felt the same emotion as the character (affective empathy). Indeed, if children felt an emotion other than that of the character but involving sadness or concern for the other person, this was sympathy, according to Eisenberg and Fabes (1998). Concerning the reliability of this measure in the present study, Cronbach’s alpha varied from 0.74 to 0.81 for TD children and from 0.66 to 0.74 for children with ID.

#### *Procedure*

The Hospital-Faculty Ethics Committee of Saint-Luc-UCLouvain approved this research procedure. To proceed to recruitment, an invitation to participate was sent to the management of kindergartens, primary schools and special schools in the French-speaking area of Belgium. A brief document explaining the aims of the research project and the conditions for participation and a consent form were sent to parents who expressed an interest in participating. Children with ID and TD children matched for developmental age were then assessed at school or at home, with two performance-based measures of their empathic skills and cognitive skills being used to determine their developmental age. For these two groups of children, two empathy questionnaires (EmQue-vf and GEM-vf) were completed separately by mothers and fathers. However, for children matched for chronological age with children with ID, only the GEM-vf was completed by mothers. To thank them for their participation, children and/or parents received a small gift at the last session.

### Results

#### *Preliminary Analyses*

Missing data analysis and inter-rater evaluation are presented in Supplementary Materials.

#### *Comparative Analyses*

**Fig. 1:**
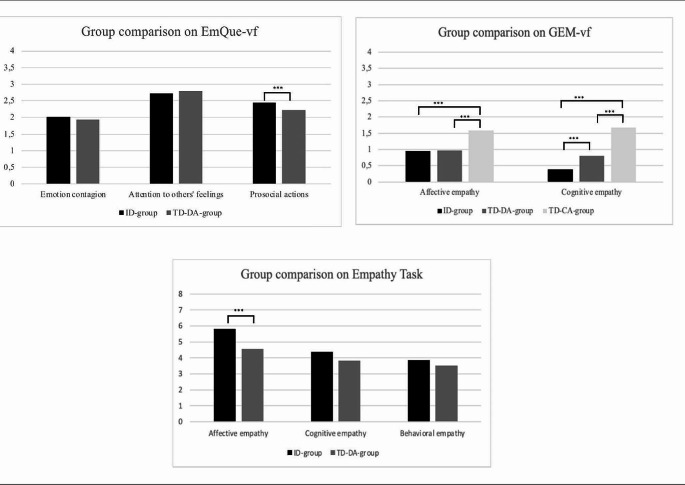
Graphs showing children’s empathy scores. *Notes* ***p < .001

Figure 1 shows the results of the comparative analyses. To investigate parents’ perception of children’s empathic skills, two one-way MANOVAs were performed. The first consisted of a comparison of the group with ID and the TD-DA group on the basis of their three subscores in the EmQue-vf (emotion contagion, attention to others’ feelings and prosocial actions). A main effect of group was obtained (Pillai’s *F* = 7.801, *p* < .001, *η*_*p*_^2^ = 0.123). Moreover, tests of between-subject found a significant difference in prosocial actions (*p* < .001), in the sense that children with ID showed more prosocial behaviors than TD-DA children (see Table 2 for means). No difference was found for emotion contagion (*p* = .132) or attention to others’ feelings (*p* = .271) between children with ID and TD-DA children. The second one-way MANOVA compared children with ID, TD-DA children, and TD-CA children in terms of their affective and cognitive empathy, as perceived by their parents through the GEM-vf. A main effect of group was found (Pillai’s *F* = 15.011, *p* < .001, *η*_*p*_^2^ = 0.110). Tests of between-subject again revealed a significant difference for affective empathy (*p* < .001) and for cognitive empathy (*p* < .001). Dunnett T3’s post-hoc analyses showed that TD-CA children were perceived as having better affective and cognitive empathy than children with ID (mean difference = 0.62 and 1.30, *p* < .001) and TD-DA children (mean difference = 0.60 and 0.89, *p* < .001). Moreover, TD-DA children were perceived as having a better understanding of others’ emotions than children with ID (mean difference = 0.41, *p* = .026), whereas these two groups were perceived as having no difference in affective empathy (mean difference = 0.018, *p* = .999)

Concerning the differences between group with ID and TD-DA group, assessed by a performance-based measure, a one-way MANOVA including affective, cognitive and behavioral scores of empathy was conducted. The results showed a group effect (Pillai’s *F* = 7.064, *p* < .001, *η*_*p*_^2^ = 0.114). Specifically, a difference between these two groups of children appeared for affective empathy (*p* < .001), in favor of children with ID (see Table 2 for means). No difference emerged for cognitive (*p* = .138) and behavioral (*p* = .375) empathy

## Study 2

### Method

#### *Objective*

The second study compared empathic skills of children with DS and children with non-specific ID with results of TD children matched for developmental or chronological age, in order to establish whether the etiology of ID is one of the factors in differences between empathy profiles. Both developmental and multi-dimensional approaches were also used in the present study. In view of the findings of the literature and the results of the first study, it was hypothesized that, for children with DS and nonspecific ID, a developmental delay in terms of their emotion contagion, attention to others’ feelings and affective empathy would be observed, compared to TD-DA children, but that they would be found to have less affective empathy than TD-CA children. Moreover, it was expected children with DS would show more prosocial behaviors than children with nonspecific ID and TD-DA children. In terms of cognitive empathy, a developmental delay was expected for children with DS and nonspecific ID. Moreover, it was expected that children with DS as well as children with nonspecific ID would be perceived as having lower skills than TD-CA children.

#### *Participants*

In this second study, four groups of children participated. The first consisted of 23 children with DS (9 girls and 14 boys), aged 5 to 14 years, with a developmental age range from 33 to 75 months (see Table 4 for means). These children had received a diagnosis of DS with a mild to moderate ID. They were recruited based on the same criteria for inclusion (chronological age between 4 and 14 years, developmental age between 3 and 6 years, mild to moderate ID) and exclusion (absence of autism spectrum disorder). Concerning the socioeconomic status of the family, Table 5 shows the means and percentages for parents’ educational level and family income for all samples. Regarding parents’ educational level, this information was missing for 1 father. In terms of family income, the mean value of the sample corresponded to a range of 3000–3500 euros per month.

**Table 4:** Descriptive statistics of Study 2

	DS	ID	TD-DA	TD-CA		
	M (ET)	M (ET)	M (ET)	M (ET)	F	η_p_^2^
N (% of boys)	23 (60.9%)	23 (60.9%)	23 (60.9%)	23 (60.9%)		
Chronological age (in years)	10.26 (2.62)	9.19 (2.04)	3.48 (0.85)	9.88 (2.12)		
Developmental age (in years)	3.96 (0.813)	4.46 (0.742)	4.03 (0.786)	/		
Language quotient (total)	0.46 (0.12)	0.63 (0.18)	0.62 (14)	/	8.151***	0.211
Language Expression quotient	0.34 (0.16)	0.55 (0.23)	0.58 (0.20)	/	10.093***	0.243
Language Understanding quotient	0.68 (0.30)	0.74 (0.16)	0.75 (0.14)	/	0.672	0.021
***Hetero-reported measure***						
EmQue-vf - Emotion contagion (max = 4)	2.36 (0.32)	1.73 (0.38)	1.93 (0.41)	/	16.884***	0.338
EmQue-vf - Attention to others’ feelings (max = 4)	3.02 (0.33)	2.53 (0.46)	2.79 (0.31)	/	9.556***	0.225
EmQue-vf - Prosocial actions (max = 4)	2.75 (0.53)	2.12 (0.48)	2.17 (0.44)	/	11.721***	0.262
GEM-vf - Affective empathy (max = 4)	1.71 (0.53)	0.10 (1.12)	0.93 (0.87)	1.63 (1.16)	14.235***	0.322
GEM-vf - Cognitive empathy (max = 4)	0.45 (1.39)	0.24 (1.07)	0.68 (0.96)	1.72 (1.49)	10.507***	0.182
***Performance-based measure***						
Empathy task - Affective empathy (max = 8)	5.69 (1.84)	6 (2.02)	3.95 (1.79)	/	7.824***	0.192
Empathy task - Cognitive empathy (max = 8)	2.82 (2.35)	4.61 (1.99)	3.34 (2.06)	/	4.222*	0.113
Empathy task - Behavioral empathy (max = 8)	2.13 (1.93)	4.08 (2.31)	2.86 (2.05)	/	5.056**	0.113

*Notes.* DS = children with DS; ID = children with; TD-DA = TD children matched for developmental age; TD-CA = TD children matched for chronological age; *p < .05, **p < .01, ***p < .001.

**Table 5:** Demographics characteristics of study 2 participants’ family

	DS		ID		TD-DA		TD-CA	
	M (SD)	%	M (SD)	%	M (SD)	%	M (SD)	%
*Mothers’ educational level*	7.87 (1.57)		4.87 (1.95)		7.30 (1.94)		6.96 (2.01)	
≤ Vocational qualification		4.3%		40.0%		13.0%		12.0%
High school qualification		17.4%		46.7%		21.8%		24.0%
Bachelor’s degree		26.1%		6.6%		21.7%		32.0%
Master’s degree		43.5%		6.7%		34.8%		28.0%
PhD		8.7%		/		8.7%		4.0%
*Fathers’ educational level*	7.00 (2.49)		4.91 (2.07)		6.52 (2.02)		6.88 (1.94)	
≤ Vocational qualification		27.3%		45.5%		21.7%		20.8%
High school qualification		13.6%		36.4%		34.8%		25.0%
Bachelor’s degree		13.6%		9.1%		13.0%		16.7%
Master’s degree		31.9%		9.1%		30.4%		37.5%
PhD		13.6%		/		/		/
*Family income*	7.00 (2.22)		4.86 (2.62)		8.33 (2.78)		8.57 (3.13)	

The second group consisted of 23 children with a nonspecific ID (9 girls and 14 boys), aged between 5 and 12 years. Their developmental age varied between 38 months and 75 months. No difference regarding chronological age (mean difference = -1.07, *p* = .493) and developmental age (mean difference = 0.57, *p* = 1) was reported between children with nonspecific ID and DS. Children with another genetic syndrome (e.g., Williams’ or Fragile X syndromes), or an associated autism spectrum disorder were excluded. Concerning parents’ education level, 6 mothers and 4 fathers had followed special education. This information was missing for 8 mothers and 11 fathers of children with nonspecific ID. In terms of family income, the mean value of the sample corresponded to a monthly income of 1500–2000 euros.

The third group consisted of 23 TD preschoolers (9 girls and 14 boys), aged between 3 and 6 years. These children were matched for developmental age (TD-DA group) with children with nonspecific ID (mean difference = 0.50, *p* = .116) and children with DS (mean difference = 0.07, *p* = 1). Concerning family socioeconomic status, there was no missing data about parents’ educational level, and the mean family income corresponded to a range of 3500–4000 euros per month. One-way ANOVA showed that children with DS presented lower total scores in language than children with nonspecific ID and TD children (*F* = 8.151, *p <* .001, *η*_*p*_^2^ = 0.211). However, through a one-way MANOVA (Pillai’s *F* = 4.352, *p* = .002, *η*_*p*_^2^ = 0.121), a group difference was reported in language expression (*F =* 10.093, *p* < .001, *η*_*p*_^2^ = 0.243). More precisely, children with DS showed lower level of language expression than children with nonspecific ID ( *p =* .002) and TD children matched for developmental age (*p* < .001). No difference in children with nonspecific ID and TD children was reported in language expression (*p =* 1). Concerning language comprehension, no difference between the three groups of children was identified (*F =* 0.672, *p* = .514, *η*_*p*_^2^ = 0.021).

Finally, the last group consisted of 23 TD children, aged between 5 and 13 years. Those children were matched for chronological age (TD-CA group) with children with nonspecific ID (mean difference = 0.312, *p* = .846) and children with DS (mean difference = 0.312, *p* = .846). Concerning family socioeconomic status, no missing data were reported about parents’ educational level, and the mean family income corresponded to a range of 3500–4000 euros per month.

Regarding the socio-economic status of families, chi-square analysis showed that the four groups of children differed according to the level of education of mothers (χ^2^ = 40.943, df = 24, *p* = .017) and fathers (χ^2^ = 38.953, df = 21, *p* = .010), but also according to family income (χ^2^ = 62.827, df = 36, *p* = .004). Descriptive analysis showed that families of children with ID had a lower socio-economic status than families of children with DS and families of both groups of TD children.

#### *Measures and Procedure*

The measures and the procedure used in Study 2 were the same as in Study 1.

### Results

#### *Preliminary Analyses*

Missing data analysis and inter-rater evaluation are presented in Supplementary Materials.

#### *Comparative Analyses*

**Fig. 2:**
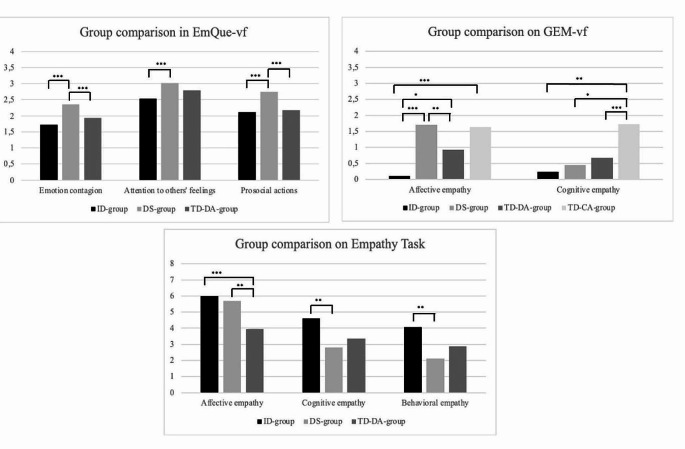
Graphs representing children’s empathy scores. *p < .05, **p < .01, ***p < .001

Figure 2 shows the results of the comparative analyses in this second study. Two one-way MANOVAs were performed to investigate group differences depending on parents’ perception of children’s empathic skills. The first consisted of a comparison of the group with ID, the group with DS, and the TD-DA group on the basis of their three subscores in the EmQue-vf (emotion contagion, attention to others’ feelings and prosocial actions). A main effect of group was demonstrated (Pillai’s *F* = 6.629, *p* < .001, *η*_*p*_^2^ = 0.234). Moreover, tests of between-subject found a significant difference in emotion contagion (*p* < .001), attention to others’ feelings (*p* < .001) and prosocial actions (*p* < .001). Bonferroni’s post-hoc highlighted that children with DS were perceived as having more emotion contagion than TD-DA children (mean difference = − 0.42, *p* = .001) and children with ID (mean difference = − 0.63, *p* < .001). Children with DS were also perceived as paying more attention to others’ feelings than children with ID (mean difference = 0.49, *p* < .001). However, TD-DA children had the same level of attention to others’ feelings as children with ID (mean difference = 0.25, *p* = .08) and children with DS (mean difference = 0.23, *p* = .117). Concerning prosocial actions, children with DS showed more prosocial behaviors than TD-DA children (mean difference = 0.57, *p* < .001) and children with nonspecific ID (mean difference = 0.63, *p* < .001). Finally, no difference between TD-DA children and children with nonspecific ID was reported (mean difference = 0.05, *p* = 1)

The second one-way MANOVA compared the group with ID, group with DS, TD-DA group, and TD-CA group of children in terms of their affective and cognitive empathy, as perceived by their parents through the GEM-vf. A main effect of group was found (Pillai’s *F* = 9.047, *p* < .001, *η*_*p*_^2^ = 0.232). Tests of between-subject again revealed a significant difference for affective empathy (*p* < .001) and for cognitive empathy (*p* < .001). Dunnett T3’s post-hoc analyses showed that children with DS and TD-CA children had equivalent affective empathic skills, as perceived by their parents (mean difference = 0.079, *p* = 1.000). Moreover, children with DS were perceived as having better affective empathy than TD-DA children (mean difference = 0.78 *p* = .003) and children with nonspecific ID (mean difference = 1.60, *p* < .001). However, children with nonspecific ID had poorer results for affective empathy than TD children matched for developmental age (mean difference = − 0.82, *p* = .044) and matched for chronological age (mean difference = -1.52, *p* < .001). Concerning cognitive empathy, children with DS (mean difference = 1.27, *p* = .022), children with nonspecific ID (mean difference = 1.04, *p* = .002), and TD-DA children (mean difference = 1.04, *p* = .003) were perceived as less understanding of others’ emotions than TD-CA children. Finally, there was no difference in terms of cognitive empathic skills between the three other groups (mean difference between 0.21 and 0.44, *p* between 0.603 and 0.986)

 To investigate the differences between the group with ID, group with DS and TD-DA group, assessed by a performance-based measure, a one-way MANOVA including affective, cognitive, and behavioral scores of empathy was conducted. The results showed a group effect (Pillai’s *F* = 7.146, *p* < .001, *η*_*p*_^2^ = 0.248). Specifically, a difference between these groups appeared for affective empathy (*p* = .001), for cognitive empathy (*p* = .019) and for behavioral empathy (*p* = .009). Bonferroni’s post-hoc highlighted that TD-DA children had poorer skills than children with DS (mean difference = -1.74, *p =* .008) and children with nonspecific ID (mean difference = -2.04, *p =* .001), while children with DS and nonspecific ID had equivalent skills (mean difference = 0.30, *p =* 1.000). Concerning cognitive empathy, children with DS had poorer results than children with nonspecific ID (mean difference = -1.78, *p* = .019). No differences between TD-DA children and children with nonspecific ID (mean difference = -1.26, *p* = .149) or children with DS (mean difference = 0.52, *p* = 1.000) were reported for cognitive empathy. A difference was found between children with nonspecific ID and children with DS in terms of their behavioral empathic skills, in favor of children with nonspecific ID (mean difference = 1.96, *p* = .007). TD-DA children had equivalent results to those of children with nonspecific ID (mean difference = -1.2, *p* = .163) and children with DS (mean difference = 0.74, *p* = .715)

### Discussion

The principal aim of these studies was to gain a better understanding of children with ID’s empathic skills with respect to a developmental and a dimensional approach to empathy. To do this, children with nonspecific ID were compared to two groups of TD children (matched for chronological or developmental age). The first study focused on understanding empathic skills of children with mild to moderate ID, while the second sought to determine whether the etiology of ID (in terms of DS/nonspecific ID) plays a role in the development of empathic skills at a preschool developmental age.

The first study revealed differing conclusions depending on the kind of measure used. In line with the findings of Sigman et al. (1992), parents of children with ID reported similar levels of affective empathy to those reported by the parents of TD-DA children. In comparison with TD-CA children, children with ID were perceived as having less affective empathy, as previously found by Dyck et al. (2001) and Merrell et al. (1992). No differences in emotion contagion and in attention to others’ feelings were reported in children with ID and TD-DA children. As expected, these results lead us to conclude that there is a developmental delay in affective empathy in children with mild to moderate ID, on the basis of parents’ perception of implicit empathy in children’s daily life. However, the performance-based measure highlighted that children with ID have better explicit affective empathy than TD-DA children. Although this result is inconsistent with the literature, which has generally found that children with ID have equivalent or less empathy than TD children, it could be explained by the kind of response given by the two groups of children. Although children with ID noticed that they felt the same emotions as the protagonist of the story, TD-DA children felt more sympathy towards the character. Sympathy is defined as an emotional response to another’s emotional state that is not identical to the other’s emotion but involves sadness or concern for the other person (Eisenberg & Fabes, 1998). TD-DA children were more likely to express concern or sadness in response to the protagonist’s anger or fear (e.g. “*She is angry because the dog has broken her doll and it makes me feel sad”*). The hypothesis about affective empathy was therefore partially confirmed.

Concerning cognitive empathy, the results of the parental questionnaire highlighted that children with ID were perceived by their parents as having less cognitive empathy in their daily lives than TD-DA children, contrary to expectations. It therefore may be difficult for children with ID to understand others’ emotions in an empathic context with complex interactions. With reference to difference hypotheses, the cognitive empathic skills children with ID appeared to be deficient, compared to TD-CA children, when their skills were assessed by parents and concerned ecological situations. However, through the stories in the empathic task, the performance of children with ID in terms of explicit cognitive empathy was comparable to that of TD-DA children, which confirms the hypothesis about cognitive empathy. This finding was close to those of studies on the understanding of emotions in research about Theory of Mind (Baurain & Nader-Grosbois, 2013; Thirion-Marissiaux & Nader-Grosbois, 2008; Wishart & Pitcairn, 2000), which is an analogous construct of cognitive empathy but concerned more with the understanding of causes and consequences of emotions. The results of these mentioned studies suggest a developmental delay in this capacity. In other words, children with ID seemed to be able to justify why they felt this emotion, elicited by the emotion of a story protagonist, as well as TD-DA children. This result could be explained by the fact that the stories told in the performance-based measure presented simple emotional situations involving a single child, unlike the items completed by parents, which referred to more complex everyday situations.

Surprisingly, although children with ID’s performance in terms of explicit behavioral empathy in the empathy task was similar to that of TD-DA children, they were perceived as displaying more prosocial actions by their parents. As they were older than the TD-DA children, the children with ID may have experienced more emotional situations, enabling them to engage in prosocial behaviors. In other words, seeing someone feel an emotion caused children with ID to spontaneously engage in comforting, helping, or sharing behaviors more than TD-DA children. Therefore, children with ID’s prosocial actions in an empathic context were qualified by a positive difference in the view of their parents, whereas a developmental delay was highlighted through the use of hypothetical stories. This finding contradicts those of the study by Zion and Jenvey (2006), which highlighted a deficit in prosocial behaviors in children with ID. However, it is important to note that, unlike the present study, children with ID were matched with TD children according to their chronological age in the study by Zion and Jenvey (2006). Moreover, that study evaluated social skills in a broad sense whereas the present study only considered prosocial behaviors.

The aim of the second study was to examine children’s empathic skills depending on the etiology of the ID. To do this, children with DS and nonspecific ID were compared to TD children matched for developmental or chronological age, using the same methodology as in the first study.

Whether measured through a parental questionnaire or a performance task, affective empathy seemed to be strongly present in children with DS. Contrary to expectations, they were perceived by their parents as having more affective empathy and they performed better in the empathy task than TD-DA children, which highlighted a positive difference in terms of the difference hypothesis. They showed the same level of affective empathy as TD-CA children when they were assessed by their parents. Compared to children with nonspecific ID, children with DS were perceived as having more affective empathy, which was consistent with the findings of Kasari et al. (1990), namely that children with DS expressed more positive emotions and were more involved in intense interactions than children with nonspecific ID. Early interventions toward children with DS (e.g., medical, linguistic, developmental, psychomotor intervention at an early age) may be more conducive to their empathic development than that of children with nonspecific ID. Indeed, being supported by professionals since childhood in their development could foster socioemotional skills and prevent behavioral problems. However, they had equivalent results in the performance-based measure. Moreover, although children with DS were perceived as having more emotion contagion than children with nonspecific ID and TD-DA children, they were also perceived as paying more attention to others’ feelings than children with nonspecific ID. Referring to Hoffman’s (1987) developmental model, children with DS seemed to present more reactions from previous development stages than children with nonspecific ID. This finding could be interpreted as indicating oscillations in difficult stage transitions, in line with Inhelder’s (1963) postulate of *viscosity of reasoning* or potential cognitive subfunctioning (Paour, 1992), or regressive behaviors (Nader-Grosbois, 2020, pp. 230–231). In addition, these results were consistent with the study of Kasari et al. (2003), which found that children with DS were more attentive to others’ faces and looked longer at the distressed adult than with nonspecific ID and TD-DA children.

As expected, children with DS were also perceived as having the same level of cognitive empathy in an empathic context as children with nonspecific ID and TD-DA children, supporting the delay hypothesis. However, parents perceived children with nonspecific ID as having equivalent cognitive empathy to TD-DA children, whereas they had lower scores in the first study. Given that the two groups’ ages were significantly lower in the second study, these results suggest that TD children may develop (socio)cognitive skills faster than children ID, who may have slower development or undergo stagnation in some areas (Nader-Grosbois, 2014, pp. 103–166). Finally, TD-DA children were perceived as having better skills in cognitive empathy than children with DS and nonspecific ID. Moreover, and contrary to expectations, children with DS seemed to have difficulties with cognitive empathy in the performance-based measure, compared to children with nonspecific ID. In line with the literature stipulating that children with DS have a delay in language development (Marchal et al., 2016; Polišenská & Kapalková, 2014), the results of this study showed that children with DS had a lower level of language expression, in comparison to children with ID and TD-DA children which have a similar level of language expression. They may therefore have had difficulties verbally expressing responses to questions referring to cognitive empathy. However, no differences between children with DS and TD-DA children were reported in the empathy task, supporting the delay hypothesis.

In line with Kasari et al. (2003), children with DS were perceived by their parents as engaging in more prosocial actions in response to others’ emotions than children with nonspecific ID and TD-DA children. Hence, an empathic context could influence children with DS to spontaneously comfort or help adults and/or children when they experience a specific emotion. This result supports expectations about implicit behavioral empathy. However, when behavioral empathy was assessed by a performance-based measure, children with DS performed at a lower level than children with nonspecific ID, which does not support the hypothesis. As with cognitive empathy, language delays or deficits may make it difficult for them to express what they would do when faced with another person’s emotion.

Although this study helps to refine knowledge about the empathic skills of children with ID, some limitations should be taken into account. First, the sample sizes of the different groups of children were small, and these questions need to be investigated in a larger sample of families from more diverse socio-cultural and socioeconomic backgrounds. In this study, families of children with ID presented a lower socioeconomic status than those of TD children’s families. However, the literature advanced that children from families with a higher socioeconomic status contributed to better socioemotional skills in children, including empathic skills (Malti et al., 2010). It would be interesting to control the environmental effect on the development of empathic skills in further studies. Secondly, although the children in the different groups were matched according to developmental level or chronological age, language differences could be identified between children with ID/DS and TD children. Although no difference in language comprehension was reported, children with ID in Study 1 or SD in Study 2 showed difficulties in language expression. Therefore, matching children according to language level could be a solution to determine whether empathy difficulties result or not from language difficulties. Second, although a performance-based measure and parental questionnaires were used, it may be interesting to use more contextual measures, such as observation in children’s daily life settings (e.g., at home or at school). Third, data collection in the group of children with ID turned out to be difficult, specifically using parental questionnaires. Missing data may have affected the results, despite the use of missing data analyses.

Although affective and behavioral empathy seem to be strongly present in children with ID (including those with DS), compared to TD-DA children, parents and professionals should be attentive to their specific empathic qualities. These children may show difficulties in understanding others’ emotions, resulting in emotion contagion in some cases. In order to help children with ID to cope with their empathy difficulties, it would be useful to teach them to recognize their own emotions as well as those of others, to understand the causes and consequences of emotions and to display social behaviors, in a more appropriate manner, in response to the emotions of others. With this in mind, some authors have encouraged the reading of stories (Kucirkova, 2019), role play (Knell, 2015) and the use of prosocial video games (Li & Zhang, 2023) or interactive digital stories (Bratitsis & Ziannas, 2015) that illustrate or simulate fictional critical social situations in which the protagonists need to show empathy towards other characters. Although these techniques were developed for TD preschoolers, it could be useful for children with ID if adapted materials to their capacities are used. Moreover, trainings which target the empathic behaviors could be elaborated according to the radical behaviorist view of empathy to foster verbal and nonverbal behaviors. In this way, children’s behaviors could be more adapted regarding the contextual index (Melton et al., 2023). Finally, to assess the extent to which these results are generalizable or more specific to subgroups of children with various syndromes/etiologies, it might be interesting to refine the risk factors (e.g., prematurity, infectious diseases, under stimulation) of children with non-specific ID to observe their strengths and weaknesses within their empathy skills. we recommended comparing the multiple dimensions of empathy in children presenting different genetic syndromes, using multi-method and multi-informant designs, in order to take into account their inter- and intra-variability.

## Electronic supplementary material

Below is the link to the electronic supplementary material.

Supplementary Material 1
