# Correction: How do Children with Intellectual Disabilities Empathize in Comparison to Typically Developing Children?

**DOI:** 10.1007/s10803-024-06648-0

**Published:** 2024-12-05

**Authors:** Poline Simon, Nathalie Nader-Grosbois

**Affiliations:** Chair Baron Frère in special education, Psychological Sciences Research Institute, UCLouvain, Louvain-la-Neuve, Belgium

**Correction: Journal of Autism and Developmental Disorders**

10.1007/s10803-024-06340-3

In this article in “Study1” participants section in the sentence starting “first one consisted of 79 children....” the word “nonspecific” is incorrect should have been removed and in the sentence starting “Children with...” the word “a genetic syndrome or” is incorrect should have been removed.

In “Study2” participants section in second paragraph before the sentence starting “concerning parents..” the missing sentence “Children with another genetic syndrome (e.g., Williams’ or Fragile X syndromes), or an associated autism spectrum disorder were excluded.” should have been included.

In “Discussion” paragraph in the sentence starting “To do this,...” the word “nonspecific” should have been removed.

The original article has been corrected.

