# Supplementary material for: How do Children with Intellectual Disabilities Empathize in Comparison to Typically Developing Children?
Source: J Autism Dev Disord. 2024 Apr 12;55(5):1754–69. doi: 10.1007/s10803-024-06340-3 (PMC12021724; doi:10.1007/s10803-024-06340-3)
Supplement: Supplementary file 1 — Supplementary Material 1 [file 10803_2024_6340_MOESM1_ESM.docx]

**Supplementary Materials – Preliminary analyses**

**Study 1**

1. *Missing data analysis*

Before the comparative analyses were conducted, the different empathy variables were verified to examine missing values, using IBM SPSS 27. For the group with ID, the percentage of missing data across EmQue-vf and GEM-vf completed by mothers and fathers separately varied from 34.2% to 54.4 %. Moreover, the pattern of missingness was completely random (Little’s MCAR test: $\chi$^2^ = 647.427, DF = 1075, *p* = 1.000). For the TD-DA group, the percentage of missing data EmQue-vf and GEM-vf completed by mothers and fathers separately varied from 13% to 38 %. Again, the pattern of missingness was completely random (Little’s MCAR test: $\chi$^2^ = 562.118, DF = 653, *p* = .996). To maximize statistical power, missing values were therefore retrieved using expectation maximization imputation in these two groups. For the Empathy Task and GEM-vf completed by parents of children paired for chronological age, there was no missing data.

1. *Inter-rater evaluation*

Inter-rater evaluation was performed to create a composite score for the parents’ questionnaires (EmQue-vf and GEM-vf). For this purpose, one-way random intraclass correlation coefficients (ICC) were calculated for the TD-DA group and group with ID. All the coefficients lay between .53 and .74 for the TD-DA group and between .51 and .81 for the group with ID. As Koo and Li (2016) stipulate that ICCs from .5 can be considered at least as moderate, it was possible to create composite scores to form an overall score representing both parents’ perceptions of empathic skills in their children.

**Study 2**

1. *Missing data analysis*

The different empathy variables were verified to examine missing values, using IBM SPSS 27. For children with DS, the percentage of missing data varied from 8.7% to 21.7% (mean = 15.95%). The pattern of missingness was completely random (Little’s MCAR test: $\chi$^2^ = 73.971, DF = 420, *p* = 1.000). For children with ID, the percentage of missing data varied from 30.4% to 52.2 % (mean = 41.3%). The pattern of missingness was completely random (Little’s MCAR test: $\chi$^2^ = 000, DF = 286, *p* = 1.000). For TD-DA children, the percentage of missing data EmQue-vf and GEM-vf completed by mothers and fathers separately varied from 4.3% to 30.4 % (mean = 15.32%). The pattern of missingness was completely random (Little’s MCAR test: $\chi$^2^ = 88.001, DF = 294, *p* = 1). Therefore, to maximize statistical power, missing values were retrieved using expectation maximization imputation. For the Empathy Task administered to the group with ID, group with DS, and TD-DA group, and for GEM-vf completed for the TD-CA group, no missing data was reported.

1. *Inter-rater evaluation*

For inter-rater evaluation, one-way random intraclass correlation coefficients (ICCs) were calculated for each parents’ questionnaire for each group of children (except for children matched for chronological age). All the coefficients lay between .86 for the group with DS .56, between .58 and .85 for the group with ID and between .64 and .78 for the TD-AD group. As Koo and Li (2016) stipulate that ICCs from .5 can be considered at least as moderate, it was possible to create composite scores to form an overall score representing both parents' perceptions of children's empathic skills.
